# Gene Amplification of Mediator Subunit 30 Redirects the MYC Transcriptional Program and Oncogenesis

**DOI:** 10.21203/rs.3.rs-4326418/v1

**Published:** 2024-05-06

**Authors:** Chunyu Jin, Linjie Zhao, Guofeng Zhao, Yujia Liu, Wubin Ma, Shenghong Ma, Likun Yao, Yuan Liu, Qiulian Wu, Huairui Yuan, Kailin Yang, Kenneth Ohgi, Jeremy N. Rich, Michael G. Rosenfeld

**Affiliations:** University of California, San Diego; UPMC Hillman Cancer Center; University of California, San Diego; University of California, San Diego; University of California, San Diego; University of California, San Diego; University of California, San Diego; University of California, San Diego; UPMC Hillman Cancer Center; UPMC Hillman Cancer Center; Cleveland Clinic; University of California, San Diego; UPMC Hillman Cancer Center; University of California, San Diego

## Abstract

Understanding the molecular mechanisms underlying tumorigenesis is crucial for developing effective cancer therapies. Here, we investigate the co-amplification of MED30 and MYC across diverse cancer types and its impact on oncogenic transcriptional programs. Transcriptional profiling of MYC and MED30 single or both overexpression/amplification revealed the over amount of MED30 lead MYC to a new transcriptional program that associate with poor prognosis. Mechanistically, MED30 overexpression/amplification recruits other Mediator components and binding of MYC to a small subset of novel genomic regulatory sites, changing the epigenetic marks and inducing the formation of new enhancers, which drive the expression of target genes crucial for cancer progression. In vivo studies in pancreatic ductal adenocarcinoma (PDAC) further validate the oncogenic potential of MED30, as its overexpression promotes tumor growth and can be attenuated by knockdown of MYC. Using another cancer type as an example, MED30 knockdown reduces tumor growth particularly in MYC high-expressed glioblastoma (GBM) cell lines. Overall, our study elucidates the critical role of MED30 overexpression in orchestrating oncogenic transcriptional programs and highlights its potential as a therapeutic target for MYC-amplified cancer.

## Introduction

Genome-wide sequencing have accumulated large amount of data on genetic variations in human disease, including cancers, which are driven by the simultaneous dysregulation of multiple genes [1–3]. The *MYC* gene, which encodes the transcription factor c-Myc, is the most frequently amplified oncogene driver in human cancers, and its elevated expression correlates with tumor aggression and poor clinical outcome The oncogenic effects of MYC depend on the transcription activity in which it binds to E-box sites as a heterodimer with MAX [4], and to serve as a global “transcription amplifier” for existing transcribed target genes, underlying the mechanism for rapid proliferation in cancer cells [5, 6]. However, despite considerable study on the actions of *MYC* overexpression with respect to tumor aggressiveness, it is not yet clear which transcriptional machinery/co-factors are involved in its recruitment and the basis through which elevated *MYC* activity reprograms cells to the cancer state. Moreover, MYC has been challenging to design drug, so targeted therapeutics designed to disrupt its transcriptional activity provide alternative approaches to target MYC function [7, 8]. Therefore, the identification of key factors that facilitate elevated MYC transcriptional activity will provide mechanistic insight and may inform therapeutic strategies.

Mediator (MED) is a 30-subunit complex that plays a central role in transcription regulation, in part by integrating regulatory signals from transcriptional factors to RNA polymerase II (Pol II)[9–15]. The MED complex regulates gene expression at multiple stages of transcription, from promoting assembly of the preinitiation complex (PIC) to facilitating efficient entry into elongation or promoter escape [9, 11, 16–19]. The complex contains three main integral modules; referred to as Head, Middle and Tail, and a dissociable Kinase module (CDK8, CycC, MED12 and MED13)[20]. The kinase module has been associated primarily with repressive functions but has also been implicated in activation of transcription (reviewed in [19]). The critical role of Mediator complex in transcriptional regulation and the observation that MED30 is amplified or overexpressed in ~ 70% of cancers exhibiting *MYC* amplification led us to explore its potential roles of MED30 in the MYC activation program. We then explored the potential synergistic effect of MED30 and MYC in augmenting transcription and shaping the oncogenic state. We investigated the influence of *MED30* gain-of-function and loss-of-function on tumor growth in pancreatic cancer and glioblastoma (GBM) and the underlying molecular mechanisms.

## Results

### MED30 and MYC co-amplification across various cancer types

Gene mutation, amplification, deletion and translocation are amongst the genetic events that can cause tumorigenesis. To implicate key factors that might lead to augmented transcriptional states linked to cancer, we investigated the potential role of altered expression of specific MED components in various cancer types. *In silico* interrogation of cBioportal cancer genomics database (https://www.cbioportal.org) with of approximately ten thousand cancer patients diagnosed with tens of cancer types revealed the majority of *MED* component variants represented gene amplification events. The high amplification rate of *MED* subunits may indicate gain-of-function effects of specific *MED* subunits contributing to oncogenesis. Although the frequency of *MED* genetic alterations varied among cancer types, alterations in MED components were relatively similar across cancer types, suggesting conserved roles of the MED complex in tumorigenesis. To interrogate the loss or gain of MED gene copies in pan-cancer, we examined the amplification and deletion frequency for each MED subunit gene in another database (GSCA) in all cancer types. The results overall consistent with observations in cBioportal, revealing that *MED30*, a gene coding for a 178 amino acid small Mediator subunit, exhibited the highest degree of amplification across the majority, if not all, cancer types with an amplification rate of > 10% in half of all cancer types. In contrast, *MED30* deletion frequency was the lowest among MED components (Fig. 1A).

The *MED30* gene locus is located in chromosome 8q24, the most commonly amplified region across multiple cancer types [21]. This locus contains numerous other genes and susceptible genetic variants associated with cancer prognosis, of which the most prominent is *MYC* [22, 23]. The *MED30* gene is ~ 10 Mb upstream from the *MYC* locus in 8q24. The short distance is likely to account for the frequent co-amplification (Fig. 1B). To explore how wide-spread of the co-amplification occurs, we looked into all types of cancer, and observed an average of 70% MED30 and MYC co-amplification in MYC amplified cases across all cancer types (Fig. 1C). Among all variations of these two genes in multiple cancer types, co-amplification was the dominant alteration, whereas deletion was rare (Figs. 1D and S1A, S1B). Consistent with the gene copy number’s correlation, MED30 and MYC gene expression level were also correlated in single cell level, revealed by 52,609 single cell RNA-seq in colorectal tumors and adjacent non-malignant colon tissue [24] (Fig. 1E). Further, *MED30* expression was higher in tumor vs. normal cells in many cancer types (Figure S1F). Collectively, genetic and gene expression data indicate that MED30 is significantly co-amplified with MYC and correlated in expression level.

### Med30 overexpression shifts the Myc transcriptional program.

Pancreatic ductal adenocarcinoma (PDAC) is a genetic disease driven by gene alterations, such as KRAS mutation, CDKN2A, TP53 and SMAD4 inactivation, as well as GATA6 and MYC amplification[25]. MYC amplification promotes pancreatic cancer progression by transcription factor activity that alters the expression of target genes involved in metabolism, hypoxia, and proliferation, and is correlated with poor outcome[26]. MYC is also a major driver for PDAC heterogeneity, in which the metastatic progression was associated with activation of MYC signaling pathways and enrichment for MYC amplifications specifically in metastatic patients[27]. Given the mortality and critical function of MYC amplification in PDAC, we chose to study cooperative action of MYC and MED30 in PDAC.

To examine the transcription regulation for Myc or MED30 single amplification versus MYC&MED30 co-amplification, we performed Precision run-on sequencing (PRO-seq) experiments in MYC-only overexpression, MED30-only overexpression and MYC&MED30 both overexpression Mia PaCa-2 cells versus non-overexpression control respectively, by Tet-on induced MYC(HAtagged), MED30 and MYC&MED30 expression plasmids integrated stable cell lines. 0.5 μg /ml Doxycycline successfully induce MYC, MED30 or MYC&MED30 overexpression (Figs. 2A and S2A), which mimic the physiological single and co-amplification situation states. Two-day induction resulted in upregulation of 209 genes for Myc overexpression, 587 genes for MED30 overexpression and 360 genes for MYC&MED30 overexpression (log2FC > 0.35, p < 0.05) and downregulation of 248 genes for MYC overexpression, 755 genes for MED30 overexpression and 432 genes for MYC&MED30 overexpression (log2FC<−0.35, p < 0.05) (Fig. 2A). The MYC upregulated genes are enriched in classical MYC target (MYC hallmark gene V1 and V2[28]), validating that induced exogenous MYC works equivalently to endogenous MYC (Fig. 2B). By comparing the regulated target genes in the three groups, we found the MYC&MED30 overexpression-regulated transcription program was distinct from either only MYC or only MED30 overexpression, actually closer to the MED30 overexpression program (Fig. 2B), which means many of the MYC + MED30 regulated genes are also similarly regulated under MED30 overexpression condition, and are less altered in the MYC-alone overexpression, despite the fact that MYC overexpression level was similar in the two groups. These data suggest that MED30 and MYC function synergistically on a subset of target genes.

Because Mediator and MYC both function primarily as transcription activators, the up-regulated transcription program is likely to be the direct target in MYC and/or MED30 overexpression. Functional pathway enrichment analysis revealed that the up-regulated genes in MYC-only overexpression involved the protein processing and ribosome biogenesis, which is one of the major functions of Myc[29], while that was not the case in MYC&MED30 co-overexpression. The top up-regulated pathway in MYC&MED30 co-overexpression was inflammation/TNF-alpha/RELA (Fig. 2C). Some well-studied oncogenes were activated in MYC&MED30 co-overexpression but not with MYC-overexpression alone. These oncogenes include IRAK2, which plays critical roles in pancreatic cancer initiation and progression[30]; HMGCL, which is critical for tumorigenesis and progression in spontaneous pancreatic cancer model in mice[31]; and the IL-23 receptor (IL-23R), which was initially identified as expressed in tumor-infiltrating immune cells and in tumor cells as well[32, 33]. The expression of IL-23R in tumor cells has been associated with a poor prognosis, possibly because IL-23 binds to its receptor and promotes the migration and invasion[34] (Fig. 2D). These genes are all found significantly upregulated with MYC&MED30 co-overexpression, but not with MYC-only overexpression. The top 30 significant upregulated genes for MYC&MED30 co-overexpression exhibit a poor prognosis signature in pancreatic cancer patients analyzed by disease-free survival, while those for MYC-only upregulated genes do not exhibit a significant effect, at lease at the current data size (GEPIA, gepia.cancer-pku.cn)(Fig. 2E). To confirm the transcriptional activity correlates with final RNA level, we also performed RNA-seq in MED30 overexpressing for two days versus non-overexpression, and observed RNA-seq results indeed correlate with the observation from PRO-seq (Figure S2B). These results suggest that the MYC&MED30 co-amplification largely shifted the transcriptional program compared to single factor amplification, with the new transcriptional program further promoting cancer progression.

### MED30 overexpression promotes MYC binding to additional genomic regulatory loci

In considering the potential mechanisms by which MYC&MED30 co-overexpression induced a distinct transcriptional program from MYC-alone, we wanted in investigate whether MED30 overexpression drives MYC binding to additional sites. To test this hypothesis, experiments involving MYC CUT&TAG were conducted following the overexpression of MED30. Assessing MYC binding upon doxycycline-induced *MED30* overexpression for 1 or 5 days, revealed ~ 4000 *de novo* binding sites after 5 days of overexpression. MYC binding was now induced at these de novo MED30 co-bound regulatory regions, which were also marked by active regulatory marks H3K27ac and/or H3K4me3 (Fig. 3A). Maintained binding peaks, which were stronger than lost or gained peaks, were not further increased with MED30 overexpression, but gained peaks were increased even after a 1-day induction (Figs. 3A and S3A). MED30 overexpression did not increase overall MYC protein levels (Fig. 3B), supporting the interpretation that MED30 enhanced recruitment of MYC binding to novel sites to which MED30 bound upon overexpression. The gained peaks were enriched around the genes changed in MED30 overexpression versus control (Figure S3B). Among the ~ 4000 gained peaks, ~ 80% were located on promoters and ~ 400 (10%) were located on enhancers within 50 kb of TSSs. To examine if promoter or enhancer binding alterations were responsible for MED30-mediated gene expression changes, we analyzed PRO-seq gene expression fold-change for gained promoter-binding or gained enhancer-binding in the transcriptome with log2FC > 0 in MED30 overexpression versus control. We observed that genes with MYC gained binding on promoter or enhancer exhibited higher induction level than those without MYC binding, which indicates that either promoters and/or enhancers that gained MYC binding were functional (Figs. 3C and S3C).

Examining the effects of MED30 overexpression on regulation of specific genes, *MED30* overexpression caused MYC binding at the *PURA-CYSTM1* locus to an intergenic enhancer between the *PURA* and *CYSTM1* promoters (Fig. 3D). Correspondingly, *PURA* and *CYSTM1* expression levels were elevated upon *MED30* overexpression (Figs. 3D,3E and S3D). *IGIP* is an intervening gene located between *PURA* and *CYSTM1*, but is inactive at baseline and is not bound by Mediator. It was not activated upon *MED30* overexpression, whereas an adjacent gene, *NRG2*, which is located in an H3K27me3-marked repressive region but which was bound by Mediator and MYC, exhibited a mild activation upon *MED30* overexpression, suggesting that the newly occupied enhancer binding sites can result in activation of existing cognate promoters (Fig. 3D). Reciprocally, knockdown of *MYC* reduced *PURA* and *CYSTM1* gene expression both at baseline and upon *MED30* overexpression, indicating that these are MYC gene targets (Fig. 3E). The *HMGCL-FUCA1* locus was regulated in a similar manner, with *MED30* overexpression inducing *de novo* binding of MYC at an intergenic enhancer of *HMGCL* and *FUCA1* and increased expression of *HMGCL* and *FUCA1* (Fig. 3D). *MYC* knockdown inhibited MED30-induced *FUCA1* upregulation (Fig. 3E). Functional significance of *MYC* recruitment was supported by the finding that *MYC* knockdown compromised the induction of target genes caused by MED30 overexpression, demonstrating that recruitment of MYC was required for activation of these target genes.

### Recruitment of MYC by MED30

We next investigated whether the additional MYC binding sites in response to MED30 overexpression reflects the direst actions of MED30, or by other indirect mechanisms. We first performed MED30 CUT&TAG with or without Dox induction for 3 days in Tet-on MED30 overexpression Mia PaCa-2 cells. The MED30 binding profile indeed effectively differentiated between MED30 overexpression and control conditions (Figure S4A). With stringent criteria (log2FC > 0.8 p < 0.05), there are 138 significantly elevated (gained) sites, and 128 of down-regulated peaks (log2FC<−0.8 p < 0.05) (Fig. 4A). We found that these MED30 increased and decreased sites were correlated with higher or lower binding of MYC (Fig. 4B), suggesting that MED30/mediator effectively licenses recruitment of MYC to these new sites. To confirm this, MED30 was knocked down using the Tet-on shMED30 knockdown system in Mia PaCa-2 cells and MYC ChIP-seq performed to test whether MED30 was required for MYC binding on the genome in normal conditions. MYC binding density was substantially reduced upon MED30 depletion, with over 5000 peaks lost upon shMED30 transduction (Fig. 4C). The median ratio for all MYC binding peaks in shMED30 vs. control was 0.56 (Figure S4B). Genome browser tracks showed the promotor binding peaks were decreased on known MYC target genes, examplified by PABPC1 and PGK1 (Figure S4C). Together, these data demonstrated that the MED30/mediator complex can recruit and/or stabilize MYC binding widely in the genome, including a large cohort of new promoter and enhancer sites.

Next, we investigated the role of MED30 in MYC-mediated target gene regulation by performing PRO-seq in Tet-on shMED30 with or without Dox treatment, with 1/50 Drosophila S2 cells were spiked-in to normalize the global effect. While the human samples clustered together by treatment, the Drosophila samples evenly distributed in all samples (Figure S4D). With > 95% of the MED30 mRNA lost by shRNA knockdown, the major portion of the Mediator complex was presumably lost[35]. This MED30 knockdown resulted in a down-regulation of 13% (n = 2695) and up-regulation of 3.2% (n = 647) of expressed genes (Fig. 4D). The genes regulated by MED30 were subjected to a hallmark gene search, revealing a notable enrichment of the Myc Target (Fig. 4E). Consensus analysis of transcription factors for the regulated genes revealed a significant enrichment in MYC or MAX binding (Fig. 4E). Notably, 106 out of 200 Myc target V1 hallmark genes were found to exhibit significantly altered regulation upon MED30 knockdown, indicating a substantial reliance of MYC transcriptional activity on MED30/Mediator (Figure S4E). KEGG pathway analyses showed the significant down-regulated pathway in MED30 knockdown condition were ribosome, which is consistent with MYC overexpression PRO-seq data as well as cell cycle, coronavirus disease, and the up-regulated pathway was steroid biosynthesis (Figure S4F).

The gene transcription levels for both MYC binding-lost and maintained groups were decreased following MED30 knockdown, while the transcription level for MYC binding -gained group with MED30 knockdown, was not altered (Fig. 4F). We noted that MYC is one of the transcriptional target genes for MED30 based on our shMED30 PRO-seq (Figure S4E), consistent with other data [36]; however, total MYC protein level was not reduced upon MED30 knockdown, possibly due to a feedback loop (Fig. 4G). These data indicated that MED30 is required for MYC-targeted transcriptional activity rather than acting to regulate MYC protein levels.

### MED30 recruit other Mediator components to the gained sites

We further investigated whether it is MED30 alone, or the Mediator complex, that mediate the gained transcription in response to MED30 overexpression. Firstly, we performed MED1 CUT&TAG with or without MED30 overexpression for two days in Mia PaCa-2 cells to determine whether MED1 was recruited to MED30 gained sites using Tet-on MED30 overexpression Mia PaCa-2 cell line. The results showed that MED1 binding was indeed corelated with that of MED30. MED30 gained sites also gained MED1, while MED30 lost sites also lost MED1 (Figs. 5A and S5A). The gained binding was indeed due to MED30 overexpression rather than Dox effect, because it was not observed in Tet-on shMED30 cell line with or without Dox induction (Figure S5B).

To expand this observation, we further performed MED4, MED12, MED17, and MED23 CUT&TAG in response to MED30 overexpression, and also included MED1 and MED30 to confirm the result. In dox-induced MED30-HA overexpression Mia PaCa-2 cells. The genome-wide analysis confirmed that MED30 gained binding sites are robustly reproducible in independent experiments. Other Mediator components, spanning Head, Middle and Tail module, including MED17 (Head module) and MED23 (Tail Module) also increase binding on MED30-gained sites (Figure S5C). The most significant upregulated MED30 peaks exhibit high levels of all other Mediator components’ binding that tested (Fig. 5B), and the gene expression adjacent to the gained peaks were significantly elevated (Fig. 5C). To further assess the requirement for MED30 in maintaining Mediator complex level in the genome, we performed Cut&Tag on MED1, MED 17, and MED 23 with or without MED30 knockdown in Mia PaCa-2 cells. Peak heat map analysis showed that the binding density of all these subunits decreased upon MED30 knockdown (Fig. 5D and S5D). Genome browser snapshots confirmed the decrease in strong binding sites in histone gene cluster regions and weak binding sites across the genome (Figure S5E). In summary, MED30, as a core Mediator subunit is essential to recruit other Mediator components to genomic sites.

### MED30 overexpression create new enhancers

We next examined whether the histone landscape changes along with the alteration of Mediator and MYC binding. We assessed the active enhancer mark H3K27ac, the repressive (poised enhancer) mark H3k27me2 and the active H3K4me3 promoter mark by CUT&TAG in Dox-induced MED30 overexpression Mia PaCa-2 cells. Genome browser tracks verified H3K4me3 indeed on gene promoter, H3K27ac on promoter and intergenic region, and H3K27me2 spread on genome but avoid of active region (Figure S6A). We then mapped the gained and lost binding sites for MED30 to these epigenetic marks, observing increased H3K27ac, decreased H3K27me2, but no change in H3K4me3 in MED30 gained sites (Fig. 6A). Reciprocally, the MED30 lost sites exhibit decreased H3K27ac, and unchanged H3K27me2 and H3K4me3 marks. The relatively low level of H3K27ac and H3K4me3 compared to the gained sites indicate that most of the lost sites were not associated with active enhancers and/or promoters (Fig. 6A). Integrated with PRO-seq with or without MED30 overexpression, we observed that these gained MED30 binding sites, which most are co-localized with enhancers or/and promoters, also gained transcriptional activity, while decreased sites exhibited low transcription activity with no significant change, consistent with the epigenomic status (Fig. 6A). Genome browser tracks showed the new MED30/Mediator complex/MYC binding sites indeed gained new generated enhancer marks (Fig. 6B). The new gained enhancers represented only a limited cohort of the whole genome-wide enhancers (Figure S6B), consistent with the small number of genes were regulated. Collectively, these data indicate that MED30 overexpression induced the level of active enhancer mark decoration on the gained binding sites, and elevated the enhancer RNA (eRNA) and target gene transcription.

### MED30 overexpression promotes pancreatic cancer cell growth in vitro and in vivo

Given the frequency of *MED30* amplification in cancer, we assessed the functional contributions of MED30 expression on tumor growth. First, we tested the effects of MED30 overexpression on Mia PaCa-2 cell growth with gradient overexpression. Dox-induced MED30 overexpression mildly increased cell growth *in vitro* (Figs. 7A). As tumor growth regulation displays critical differences *in vitro* and *in vivo*, we investigated *in vivo* MED30 growth dependence in nude mice tumor xenografts, revealing that Dox-induced MED30 overexpression promoted Mia PaCa-2 xenograft growth (Fig. 7B). To test the hypothesis that the oncogenic effect of MED30 was dependent on MYC, we used lentiviral transduction of MED30 Dox-inducible tumor cells with either a non-targeting control shRNA (shCTL) or sh*MYC*, and then tested tumor growth *in vivo*. MED30 overexpression with shCTL maintained the tumor growth acceleration effect of MED30, but targeting *MYC* compromised the growth-promoting effects of MED30, suggesting that the oncogenic effects of *MED30* are dependent on MYC (Figs. 7C-E). Reciprocally, knockdown of MED30 also dramatically reduced growth of Mia PaCa-2 cells, whereas the effects of *MED15* knockdown were minimal (Figs. 7F, S7A, and S7C), indicating the Mediator complex’s function, rather than non-essential subunit, is critical for cell growth. The effects in 293T cells were less pronounced (Figure S7B). To further characterize the phenotype of MED30 perturbation in cancer, we performed a series of cell-based assays in Mia PaCa-2 cells upon MED30 knockdown. *MED30* knockdown increased the percentage of cells displaying DNA damage, based on immunostaining of the double strand DNA break marker, γH2AX (Figure S7D). MED30 knockdown increased tumor cell apoptosis as measured by Annexin V staining (Figure S7E). *In vivo*, we found *MED30* knockdown reduced pancreatic cancer (Mia PaCa-2) xenograft growth in nude mice (Figs. 7G, 7H, and 7I). Together, these results indicate the role of MED30 in pancreatic cancer cells survival and growth.

Given the oncogenic roles of MED30 at the molecular and cellular level, we interrogated MED30 status with cancer outcome in pancreatic cancer patients. High *MED30* expression levels correlated with poor overall survival in Pancreatic adenocarcinoma (PAAD) patients (Fig. 7J). We next analyzed the survival associated with amplifications of either *MYC* or *MED30* or co-amplification from the ATCC database. Although the tumor case numbers harboring amplifications of either *MYC* or MED30-only amplification was too low to draw highly significant conclusions, we do observe both amplifications significantly decrease the survival probability (Fig. 7K).

### MED30 and MYC co-amplification functions in multiple cancer types

Because MED30 gene amplification is found in almost all cancer types, we examined MED30 function in another highly malignant cancer type – brain cancer[37]. Consistent with the co-amplification described before, MED30 and MYC gene expression are significantly correlated in all subtype of brain tumor: classical, mesenchymal and pro-neural (Fig. 8A), and in most of the histological types (Figure S8A). Glioblastoma(GBM) is a major subtype of brain cancer, and contains self-renewing, tumor-initiating glioblastoma stem cells (GSCs)[38]. Mediator components expression levels in the glioblastoma TCGA patient database analyzed by RNA-seq showed a positive correlation of MED30 with the disease grade (Figure S8B). To investigate whether GSCs proliferation and stemness are dependent on MED30, and assess the correlation with MYC levels, we employed a panel of patient-derived GSCs (GSC2907, GSC3028, GSC3565, and GSC28), including cells with various levels of *MYC* expression (Fig. 8B). We had previously demonstrated that glioblastoma stem cells are dependent on MYC function for cellular proliferation and self-renewal [39]. MED15 was used as a control of non-critical subunit of Mediator complex that does not affected other Mediator subunits’ levels by knockdown. In cell proliferation assays, knockdown of *MED30* decreased the growth of these cells to a much greater degree than upon *MED15* knockdown (Fig. 8C). The growth inhibition effect of MED30 knockdown in cells expressing high levels of MYC (GSC3565 and GSC28) was greater than in cells with lower MYC expression (GSC2907 and GSC3028), suggesting that tumor cells with high levels of MYC are more sensitive to MED30 depletion (Fig. 8C). MED30 perturbation reduced neurosphere formation GSC3565 and GSC28 cells, an assay that indicate stemness (Figs. 8D and 8E). To examine the transcription changes, GSC3565 cells transduced with or without sh*MED30* underwent RNA-seq analysis. The effects of two sh*MED30*s were quite similar with high knockdown efficacy (~ 90%) and clustering together in sample-to-sample distance analysis (Figure S8C). The number of differentially regulated genes after 2 days of knockdown was high, with ~ 2,300 downregulated and 2,400 upregulated shared genes for the two shRNAs (Figure S8D). The downregulated genes were enriched in basic cellular processes, such as ribosomal protein, electron transport, and oxidative phosphorylation, reflecting expression of the large number of altered genes (Figure S8E). These data revealed that MED30 depletion dramatically altered the transcriptome. Consistent with the result in Mia PaCa-2 cells, the transcriptome alteration was also enriched in MYC target by GSEA analysis (Fig. 8F), and top consensus TFs enriched in down-regulated gene set also include MYC (Fig. 8G).

To confirm the MED30’s function in vivo, we orthotopically implanted GSC3565 or GSC28 cells containing shCtl, or two shMED30 shRNAs into brains of nude mice brain. We found that knockdown of MED30 reduced glioblastoma tumor growth and extended lifespan of tumor-bearing mice significantly (Figs. 8H and 8I). In adult brain tumor patients with all tumor types (primary, recurrent, secondary) and all histology (oligodendroglia, oligoastrocytoma, astrocytoma, anaplastic oligodendrolgioma, anaplstic oligoastrocytoma, anaplastic astrocytoma, GBM), high MED30 levels correlate with poor survival rate (Fig. 8J).

Given that MED30 and MYC are ubiquitous expressed among tissues (Figure S8F), we speculate similar pan-cancer mechanism exists. Analysis of pan-cancer of whole genome (ICGC/TCGA, Nature 2020) in the cBioPortal database revealed that MED30 and/or MYC amplification significantly reduce the survival rate in pan-cancer patients (Fig. 8K). In summary, the phenomena and mechanisms we described in current study are likely to apply to many cancer types.

## Discussion

Despite decades of research, mechanisms mediating MYC function in cancer, with the connection to levels of the Mediator complex, remain in need of further investigation. Here, we define a novel connection between MYC and the Mediator subunit, MED30, revealed by gene amplification data, which has uncovered an overlooked aspect of the MYC transcriptional and oncogenic program and the quantitative role of levels of Mediator in specific gene activation programs. MYC and MED30 are located in relative genomic proximity (chromosome 8 q24.21 and q24.11, respectively) and are frequently co-amplified. We find a previously unappreciated mechanism in which the MED30/Mediator complex directs MYC recruitment to regulatory loci and drives oncogenic progression, eliciting the functional link of the two genetically prevalent variation events. These findings have implications for the broader issues of co-occurrence of amplification or deletion in the genome, including genes which have not yet been recognized as drivers, perhaps acting as neglected risk factors for the driver’s oncogene activity.

### A component of the Mediator complex is critical for MYC recruitment and function

The epidemiological correlation between amplification of a subunit of the Mediator complex (MED30) and cancer provides several insights into both the molecular mechanism by which increased expression of MED30 causes cancer, and uncovered a central role of MED30 in Mediator function. MED30 overexpression increased levels of the Mediator complex on a relatively limited number of promoters and enhancers that had previously not been bound by either factor, and MED30 augmented recruitment of MYC to these additional MED30-bound regulatory elements, thereby expanding the repertoire of MYC-occupied enhancers/promoters. Targeting MYC knockdown abrogates the augmentation of the MED30-induced transcriptional program, both in culture and *in vivo*. Without MED30, MYC genome binding density was largely decreased. MED30, as a core protein of the Mediator complex, critically maintains the protein stability of a cohort of mediator subunits[35], and hence in the maintenance of transcriptional programs. Cancers arises from genetic alterations that lead to dysregulated transcription programs[40], and the overexpression of MED30 provides a strong example where gain of activity of a specific component of the transcriptional coactivator machinery promotes cancer initiation and progression.

### MED30 is one of the highest genetic amplified genes in pan-caner and is a potential therapeutic target

Tens of thousands of patient sample profiling data accumulated in database showed that MED30 gene amplification is present at high level in widespread of cancer types. In cancer of breast, liver, ovary, uvea or uterus, the homozygous amplification rate is around or above 20%. This variation frequency on average is lower than TP53 mutation, similar to MYC and higher than other well-known oncogenes, for example PTEN, KRAS, PIK3CA, et al, suggesting that MED30 may serve as a biomarker for cancer subtypes, which are potentially regulated by MED30 targeting. Taking pancreatic cancer cell line and patient-derived glioblastoma cell cultures harboring high MYC expression as examples, we found that the similar transcriptome alteration in MED30 knockdown condition corresponding to the MYC knockdown signature, consistent with the cooperative function of MED30 and MYC was conserved among tissues.

Oncogenic transcription factors dictate aberrant/augmented transcriptional programs which supports rapid cell division in cancer cells. This dependency creates the vulnerability for targeting transcription as a therapeutic strategy. Indeed, transformed cells frequently display preferential susceptibility to apoptosis in response to transcriptional inhibition [41]. For example, in steroid hormone-dependent cancers, such as breast, prostate, and ovarian cancer, steroid hormone receptors and coactivators that support the nuclear receptor transcriptional programs are considered to be therapeutic targets [42, 43]. Here, we provide mechanistic insight into how co-amplification of *MED30* in ~ 70% of cancers harboring its amplification augments the transcriptional, oncogenic program mediated by MYC. Even in cells harboring amplification of only *MED30*, there is an accompanying augmented recruitment of MYC on a specific additional MED30-bound regulatory regions, suggesting that reducing *Med30* levels represents a potential therapeutic strategy. Targeting *Med30* impairs MYC function even in the absence of amplification of either protein. Thus, MED30 critically dictates the recruitment of MYC to regulatory sites in the genome. MYC binds as a homodimer or heterodimer to its DNA binding elements, referred to a E-boxes ([44, 45], as reviewed in [46]), and the absence of MAX augments binding despite increasing oncogenesis in B cells [47]. Our study reveals that MYC homo- or hetero-dimer binding to the cognate E-box sites on promoters and enhancers is increased based of the ability of MED30 to interact with MYC. Establishing the interactions of MED30 and MYC establish the role of MED30 in the oncogenic actions of MYC. Thus, in cases of isolated MED30 or MYC amplification, or even in the absence of their amplification, MED30 is a critical determinant of the program by which MYC activates growth of multiple tumor types and implies the potential therapeutic benefit of inhibition of Med30 levels or the interaction with other Mediator subunits. Given the high number of cancer patients harboring MYC and/or MED30 amplification, targeting MED30 might have wide application in antagonizing cancer progression.

Therefore, increasing levels of a specific coactivator, in this case MED30, caused a limited cohort of regulatory elements to be activated, requiring binding of a specific DNA-binding transcription factor, MYC. This suggests that limited number of TFs will be affected by such coactivator overexpression, in this case, the limited activation program increases the risk of cancer progression.

### EXPERIMENTAL MODEL AND SUBJECT DETAILS

#### Human cell lines and culture

Mia PaCa-2, HEK293T cells were obtained from ATCC were cultured in DMEM (GIBCO_#10566) media supplemented with 10% FBS. Cell lines were maintained using standard tissue culture techniques. All cells were cultured in a 5% CO2 humidified incubator at 37°C. Mycoplasma negativity was ensured routinely.

#### Animal models

NSG (NOD.Cg-Prkdcscid Il2rgtm1Wjl/SzJ) mice were purchased from the Jackson Laboratory, Bar Harbor, ME, USA. All animal experiments were approved by the Institutional Animal Care and Use Committee of UCSD.

### METHOD DETAILS

#### Construct, Lentivirus Packaging, and Stable cell line generation

pLKO lentiviral shRNA constructs and control shRNA constructs were purchased from Sigma (See Table S2). Teton shRNA constructs were homemade by annealing the shRNA sequence to pLKO.1 vector (addgene#326721). Knockdown experiments with lentivirus shRNAs were conducted according to the standard lentivirus package and transduction protocols from Addgene or Sigma. For cDNA overexpression experiments, MED30 or Myc cDNA was cloned into pTEPRT vector, and For ChIP experiment, the constructs were integrated with C-terminal or N-terminal 3XHA tag. This vector is doxycycline-inducible all-in-one construct with improved tetracycline controlled transactivator (TetR) and target gene expression driven by tandem Tet operators. The constructs carry puromycin resistant gene for positive selection. For the induction of target gene expression, titrated doxycycline was supplemented into the culture medium to achieve an expression of near endogenous level. pLKO-based lentiviral shRNA plasmids were co-transfected with packaging plasmids (psPAX2 and pMD2.G) into 293T cells. Lentiviruses were harvested, concentrated, and used for cell infection. Stable knockdown Mia PaCa-2 cells were selected with 0.4 mg/ml puromycin and collected for experiments within 5 days. For doxycycline induced knockdown or overexpression, the concentration of doxycycline was 0.5ug/ml unless specified.

#### qPCR, Data Analysis, and Statistical Analysis

For qRT-PCR experiments, the Mia PaCa-2 or glioblastoma cells with indicated shRNA knockdown or gene overeverexpression were collected RNA was isolated using RNA extraction kit (Zymo Research) or RNeasy column (QIAGEN). Total RNA then was reverse-transcribed using SuperScript III Reverse Transcriptase(Life Technologies) or following manufacturer’s instructions. qPCRs were performed in MX3000P (Stratagene) using 2X qPCR master mix from Affymetrix or Bio-Rad. Relative quantities (RQ) of gene expression levels were normalized to GAPDH unless specified. A list of primers used for qPCR is provided (Key Resources Table).

For all qRT-PCRs, experiments were performed with at least three independent biological replicates and three technical replicates for each reaction. Results are reported as mean ± SD of a representative batch Data were analyzed and statistics were performed using unpaired two-tailed Student’s t tests. Significant differences between two groups were noted by asterisks (* p < 0.05, ** p < 0.01, *** p < 0.001).

#### RNA-seq and analysis

To examine the transcriptome influence of depleting difference mediator subunit, shRNAs target MED1,MED20, MED30 or non-target Ctrl were packaged into lenti-virus and infected into Mia PaCa-2 cells, and total RNAs were extracted two days post infection. MED30 wild-type or mutant overexpression comparing to knockdown RNA seq in Mia PaCa-2 was performed with 0.5ug/ml doxycycline-induced teton shRNA or cDNA overexpression, and total RNAs were extracted two days post Dox induction. All shRNAs were packaged in lentivirus and infected into cells. RNA was extracted by Trizol (Life Technologies) RNA concentration and quality was quantified by Qubit and Agilent 2100 Bioanalyzer system, respectively. Libraries were generated using illunima PolyA RNA library kit and sequenced by HiSeq4000 with single-end reads or NovaSeq 6000 with 100 bp paired-end reads.

Following sequencing, quality control with FastQC and trimming of adaptor sequence and poly-A tails with Trim Galore, reads were mapped and assigned to the human transcripts using HISAT2. The counts file was generated by “featureCounts” on Galaxy (usegalaxy.org) with build-in genome hg38. Differential expression gene (DEG) analysis was performed using the DESeq2 package in Galaxy. Perimeters for different expression cutoff were indicated in figure legend. Functional analysis of differential expressed genes were performed on Enrichr (https://maayanlab.cloud/Enrichr/) and GSEA[1] H: hallmark gene sets.

#### Chromatin immunoprecipitation and sequencing (ChIP-seq) and analysis

To map the binding sites of Mediator component MED17, MED30 and MYC, these factors ChIP-seq were performed in Mia PaCa-2 cells with antibodies MED17 (invitrogen PA5–30314), MED30 (from Dr. Thomas G. Boyer, UT health San Antonio, homemade) and Myc (CST #9402), respectively. To test the effect of MED30 knockdown in Myc’s binding, MYC ChIP-seq was perform with or without three days 0.5ug/ml Dox treatment in Dox-induced shMED30 Mia PaCa-2 cells. To test the domain/motif contribution to binding for Myc, letivrus vector expressing HA-tagged Myc fragment and WT in Dox-induced manner were integrated into Mia PaCa-2 cells, and 0.5ug/ml Dox was administrated for 1 day, another WT sample was not treated with Dox, serving as negative control.1–5 million cells were dual cross-linked with 2 mM disuccinimidyl glutarate (DSG, ProteoChem, #C1104–1GM) for 20min at room temperature and followed by 1% formaldehyde for 10 min for Mediator’s ChIP. For transcription factors’ ChIP, cells were only fixed in 1% formaldehyde for 10 min. In both situations, the cross-linking was quenched with 0.125M glycine for 5 min. Chromatin was fragmented using a Bioruptor to get 200–500bp fragments. Subsequently, the soluble chromatin was incubated with 1–3 mg antibodies at 4°C overnight.

Immunoprecipitated complexes were collected using 20 ul Protein G Dynabeads (Life Technologies) per reaction. After washing, the protein-DNA complexes were eluted and de-crosslinked overnight at 65°C. and DNA was purified with QIAquick PCR Purification Kit (Qiagen, # 28104). The ChIP-seq DNA library construction was performed using Illumina’s ChIP-seq Sample prep kit or ABclonal’s the Rapid DNA Lib Prep Kit (ABclonal, #RK20200). Library was usually amplified by 14 cycles of qPCR. Constructed libraries were further double size selected between 150–600 bp with AMPure XP (Beckman, #A63881) and sequenced on NovaSeq 6000 with 100 bp paired-end reads at the UCSD IGM Genomics Center. The sequence tag returned by the Illumina Pipeline was adapter trimmed by Trim Galore and aligned to the hg38 assembly by Bowtie2. For subsequent visualization of ChIP-Seq signal, bigwig files were generated using the DeepTools “bamCoverage” script integrated in Galaxy server using “Normalized to reads per kilobase per million(RPKM)”. DeepTools “computeMatrix”, “plotHeatmap”, and “plotProfile” functions were used to generate heatmaps and profile plots. The data were visualized by preparing custom tracks on the University of California, Santa Cruz (UCSC) genome browser. Peaks were identified by MACS2. Peak file was uploaded to UCSC genome browser aligned with BamCoverage (bigwig) to check the reliability of peak calling. Myc ChIP for Dox-induced shMED30 was called by q < 0.1 in MACS2; others were called in default parameters. Motif analysis was performed using HOMER software package (http://homer.salk.edu/homer/). Annotation of ChIP-Seq peaks was performed using R package “ChIPseeker” with “hg38.refseq.gtf.gz” from UCSC Genome Browser as reference.

#### CUT&Tag-seq and analysis

Bench top CUT&Tag version 3 was performed as previously described[2], with modifications. Specifically, cells were seeded in 24-well plate at 30–50% confluency, and treated with indicated doxycycline concentration for two days to allow the induction of shRNAs or cDNA plasmid, and the attachment to the well’s bottom. On the day of experiment, cells in culture media were lightly fixed (0.1% formaldehyde for 2 minutes) for non-histone epitopes or without fixation for histone marks, and quenched glycine of > twice molar concentration of formaldehyde. Next, the cells were washed with PEX buffer (PBS, 2mM EDTA, 0.1% Triton X-100) for 5min thrice, in order to complete remove Mg2 + and permeabilize the cell. Then the cells were blocked by antibody buffer (0.1%BSA, 2mMEDTA) for 10–30min at room temperature, and subjected to primary antibody incubation overnight at 4°C. The antibodies were used as 1:1000 dilution in antibody buffer if not specified. The next day the primary antibody was removed and wash with wash buffer (20mMTrisCl pH7.4, 150mMNaCl, 0.05%Triton X-100) for 5min, and then incubated with secondary antibody 1:2000 diluted in wash buffer for 1h. The secondary antibody contains fluorophore (Alexa Fluor^™^) so the signal can be checked under microscope. After secondary antibody incubation, cells were washed three times with wash buffer, and then add adapter loaded pAG-Tn5 (purified in-house [3]) in Dig-300 buffer (20mMTrisCl pH7.4, 300mMNaCl, 0.05%Triton X-100) for 1h, followed by three time wash in Dig-300 buffer. Then cells were subjected to tagmentation buffer (10mMMgCl_2_ in Dig-300 buffer) at 37°C for 1–3h, and terminated and de-crosslinked by adding 7.5ul 0.5MEDTA, 8ul 10%SDS, 0.25ul 20mg/ml proteinase K per 150ul tagmentation buffer in one well and incubated at 65°C for 30 min, and transferred into Eppendorf tube to avoid evaporation and continue de-crosslinking overnight, followed by DNA purification with DNA purification Kit (Qiagen). The DNA was eluted and utilized as template for PCR reaction with i7 and i5 dual-index for library construction and sequenced on the NovaSeq 6000 with 100 bp paired-end reads at the UCSD IGM Genomics Center. Because the sequencing is deep, the signal is not sparse. The data analysis procedures were similar with in ChIP-seq above. The adapters were trimmed by Trim Galore and aligned to the hg38 assembly by Bowtie2. bigwig files were generated using the DeepTools “BamCoverage” script integrated in Galaxy server by normalizing to CPM (counts per million) or RPKM), and uploaded to UCSC genome browser for visualization. Peaks were called by MACS2 with default parameter.

#### PRO-seq and analysis

For overexpression experiment, PRO-seq was performed in doxycycline (Dox) inducible MED30, MYC-3xHA or MED30&MYC-3xHA double integrated Mia PaCa-2 cell line with or without 0.5μg/ml Dox treatment for 1 day. Samples with Dox treatment were labeled as OE (overexpression). For knockdown experiment, Dox-induced shMED30 Mia PaCa-2 stable cell line were treated or untreated with 0.5μg/ml doxycycline for 3 days before nuclei isolation. 1/50 number of *Drosophila* S2 cells were spike-in for overall input control. Each condition has two replicates. Precision run-on sequencing (PRO-seq) experiments were performed as previously described [4]. For nuclei isolation, cells were incubated with swelling buffer (10 mM Tris-Cl pH 7.5, 2 mM MgCl2 and 3 mM CaCl2) for 5 min on ice and then incubated with lysis buffer (swelling buffer with 0.5% NP-40 and 10% glycerol) for 5 min on ice, before being re-suspended in 100 μl of freezing buffer (50 mM Tris-Cl pH 8.0, 40% glycerol, 5 mM MgCl2 and 0.1 mM EDTA). For the run-on assay, an equal volume of reaction buffer (10 mM Tris-Cl pH 8.0, 5 mM MgCl2, 300 mM KCl, 1 mM dithiothreitol, 20 units of SUPERase•In, 1% sarkosyl, and 500 μM ATP, GTP, bio-UTP and bio-CTP) was added into each sample before incubation at 30°C for 5 min. The nuclear run-on RNA was then extracted with TRIzol LS reagent (10296010, Invitrogen) and subjected to hydrolysis, buffer exchange and purification by streptavidin beads (88816, Thermo Fisher). Purified RNA was treated with PNK before being used for complementary DNA synthesis by using the NEBNext Multiplex Small RNA Library Prep Set for Illumina Kit (E7300S, NEB). Obtained complementary DNA template was amplified by PCR using the Phusion High-Fidelity enzyme (M0530L, NEB) for deep sequencing.

The sequencing reads were trimed by Trim Galore. The adapter sequence to be trimed was selected as “automatic detection” for the first round and the “Illumina small adapters” for the second round(Galaxy Version 0.6.7 + galaxy0). Then the reads were mapped to hg38 Refseq database by using Bowtie2 and counted over the entire gene body (transcript) on the sense strand with respect to the gene orientation by using Featurecounts (Galaxy Version 2.0.3 + galaxy1). Deseq2 (Galaxy Version 2.11.40.8 + galaxy0) was then used to compute the significance of the differential gene expression. For the experiments with Drosophila S2 cells spike-in, data were aligned to human genome hg38 and *Drosophila* genome dm3 by Bowtie2. Gene expression counts were calculated by analyzeRepeats.pl (http://homer.ucsd.edu/homer/ngs/analyzeRNA.html), and subjected to Deseq2 analysis for identifying different expressed genes and size factor. The final Deseq2 result was calculated using the size factor for *Drosophila* samples.

#### Western blot and immunoprecipitation

Immunoblot was performed per a general western-blot protocol (Abcam). Primary antibodies were listed in Table.

For immunoprecipitations, cells were rinsed with ice-cold PBS and then lysed in mild lysis buffer (150 mM NaCl, 20 mM Tris-Cl pH7.4, 0.5% Triton X-100) with protease inhibitors (Roche, #11873580001), rotating at 4°C for 30 minutes and centrifuged at 12,000 rpm for 10 minutes. Primary antibodies were added into the supernatant cell lysate and incubated overnight 4°C with rotation. Then 20ul (for 2ug antibody) Protein A/G magnetic beads (Pierce, #88803) were added and incubated with rotation for 3 hours at 4°C. Immunoprecipitants were washed four times with lysis buffer and were denatured by SDS-PAGE sample buffer and boiled for 5 minutes. Sample were followed by immunoblot analysis with antibodies indicated in the figures.

#### Transcription/translation/proteasome inhibitor treatment

Teton Dox-induced MED30 overexpression stable Mia PaCa-2 cells were seeded into 12-well plate and added or did not add 0.5ug/ml doxycycline for 2 days before treating with indicated inhibitors. Transcription inhibitor actinomycin D (ActD) dissolved in DMSO to a concentration of 10mg/ml, translation inhibitor cycloheximide (CHX) dissolved in DMSO to a concentration of 10mg/ml, proteasome inhibitor MG-132 dissolved in DMSO to a concentration of 10mg/ml, and DMSO (vehicle control) were added to medium at 1:1000, and 1,6-HD dissolved in water was added to medium at final concentration of 2%. Cells were incubated for 8h before subjected to western blot.

#### Cell proliferation assays

Proliferation assay for Mia PaCa-2 cells was performed in two ways: one is violet staining; the other is measured by MTT (Sigma) reagent. 3000–6000 cells per well with indicated treatment were seeded in 96-well plate with 6 replicates. Violet staining: At the day of assay, cells were rinsed by PBS and fixed by 4% paraformaldehyde for 15min at room temperature before staining with 0.1% crystal violet (Sigma-Aldrich) (dissolved in 2% ethanol) for 20min at room temperature. Then cells were washed by water for 3 times followed by imaging. MTT assay: MTT was dissolved in PBS at 5mg/ml (10X working solution) and added 10ul to 100ul culture medium and incubated for 3h in the cell culture incubator. After incubation, supernatant was discarded and the plate was scanned for taking picture. Then use 100ul DMSO to dissolve the dark blue product generated in the cell, and 540nm absorbance was measured after a few minutes’ shaking.

#### Tumor xenograft models

For intracranial tumour xenograft, GSCs (10^4^/mouse, 20 μl of Neurobasal media with no Matrigel) were intracranially implanted into NSG mice (NOD.Cg-Prkdcscid Il2rgtm1Wjl/SzJ, The Jackson Laboratory, Bar Harbor, ME, USA). Mouse brains implanted with GSCs which were labeled with firefly luciferase were monitored by the bioluminescent imaging at the 4th week post injection. Animals were treated with D-Luciferin (120mg/kg, Biosynth Carbosynth, no. L-8220) intraperitoneally and anesthetized with isoflurane for the imaging analysis. The bioluminescent images were captured by an IVIS imaging system (Spectrum CT, PerkinElmer).

For Mia PaCa-2 MED30 overexpression xenograft, doxycycline-induced MED30 construct was integrated into Mia PaCa-2 cells to establish stable cell line. Lentivirus shControl or shMyc was infected into cells two days before injection. For Mia PaCa-2 shMED30 knockdown xenograft, cells were infected with lentivirus shControl or two shMED30 respectively two days before injection. Then NSG mice (NOD.Cg-Prkdcscid Il2rgtm1Wjl/SzJ, The Jackson Laboratory, Bar Harbor, ME, USA) were implanted with 10^6 cells by subcutaneous injection, and random divide into Dox and non-Dox group (n = 5 each). Dox group was supplied with 1mg/ml doxycycline in drinking water in three days post injection. Tumour dimensions were measured once when tumours were palpable. Tumour volumes were monitored twice a week till calculated using the equation (length^2^ × width)/2.

1. Subramanian, A., et al., *Gene set enrichment analysis: a knowledge-based approach for interpreting genome-wide expression profiles*. Proceedings of the National Academy of Sciences of the United States of America, 2005. 102(43): p. 15545–50.
2. Kaya-Okur, H.S., et al., *CUT&Tag for efficient epigenomic profiling of small samples and single cells*. Nature communications, 2019. 10(1): p. 1930.
3. Ma, S., et al., *Transcriptional repression of estrogen receptor alpha by YAP reveals the Hippo pathway as therapeutic target for ER(+) breast cancer*. Nature communications, 2022. 13(1): p. 1061.
4. Mahat, D.B., et al., *Base-pair-resolution genome-wide mapping of active RNA polymerases using precision nuclear run-on (PRO-seq).* Nature protocols, 2016. 11(8): p. 1455–76.
